# Segmentation of corpus callosum using diffusion tensor imaging: validation in patients with glioblastoma

**DOI:** 10.1186/1471-2342-12-10

**Published:** 2012-05-16

**Authors:** Mohammad-Reza Nazem-Zadeh, Sona Saksena, Abbas Babajani-Fermi, Quan Jiang, Hamid Soltanian-Zadeh, Mark Rosenblum, Tom Mikkelsen, Rajan Jain

**Affiliations:** 1 Control and Intelligent Processing Center of Excellence, School of Electrical and Computer Engineering, University of Tehran, Tehran 14399, Iran; 2 Department of Radiation Oncology and Radiology, University of Michigan, Ann Arbor, MI 48109-0010, USA; 3 Department of Neurology, Henry Ford Health System, Detroit, MI 48202, USA; 4 Department of Radiology, Henry Ford Health System, Detroit MI 48202, USA; 5 Washington University School of Medicine, Mallinckrodt Institute of Radiology, St. Louis MO 63110, USA; 6 Department of Radiology, Wayne State University, Detroit, MI 48202, USA; 7 Department of Neurosurgery, Henry Ford Health System, Detroit, MI 48202, USA

**Keywords:** Corpus callosum, Fiber bundle segmentation, Level-set, Glioblastoma, Diffusion tensor imaging

## Abstract

**Background:**

This paper presents a three-dimensional (3D) method for segmenting corpus callosum in normal subjects and brain cancer patients with glioblastoma.

**Methods:**

Nineteen patients with histologically confirmed treatment naïve glioblastoma and eleven normal control subjects underwent DTI on a 3T scanner. Based on the information inherent in diffusion tensors, a similarity measure was proposed and used in the proposed algorithm. In this algorithm, diffusion pattern of corpus callosum was used as prior information. Subsequently, corpus callosum was automatically divided into Witelson subdivisions. We simulated the potential rotation of corpus callosum under tumor pressure and studied the reproducibility of the proposed segmentation method in such cases.

**Results:**

Dice coefficients, estimated to compare automatic and manual segmentation results for Witelson subdivisions, ranged from 94% to 98% for control subjects and from 81% to 95% for tumor patients, illustrating closeness of automatic and manual segmentations. Studying the effect of corpus callosum rotation by different Euler angles showed that although segmentation results were more sensitive to azimuth and elevation than skew, rotations caused by brain tumors do not have major effects on the segmentation results.

**Conclusions:**

The proposed method and similarity measure segment corpus callosum by propagating a hyper-surface inside the structure (resulting in high sensitivity), without penetrating into neighboring fiber bundles (resulting in high specificity).

## Background

Corpus callosum is the largest inter-hemispheric fiber bundle in the human brain [1,2]. Most of the fibers interconnect homologue cortical areas in roughly mirror image sites but a large number of the fibers have heterotypic connections ending in asymmetrical areas [3]. Previous studies have mainly investigated effects of various pathologies on the corpus callosum [4-7]. However, a fully automated, fast, and accurate method for segmenting corpus callosum without penetrating into irrelevant neighboring structures, using data acquired in routine clinical protocols, is still lacking.

Previously, image processing methods have been proposed for segmenting corpus callosum in anatomical magnetic resonance images (MRI) [8-10]. These methods rely on intensity information of two-dimensional images and their results may need pruning. Recently, attention has been oriented towards diffusion tensor imaging (DTI) to segment white matter tracts of the brain [11,12]. Although the tensor model fails to describe higher order anisotropies in heterogeneous areas where more than one fiber population exists, it is practically useful for extracting major white matter tracts, particularly the ones with predominant diffusivity pattern such as corpus callosum. When using DTI data, the fiber bundles can be extracted by: a) clustering of fibers resulting from tractography into fiber bundles [13-19]; or b) segmenting fiber bundles via hyper-surface propagation based on local properties of diffusion tensor, diffusion signal, or orientation distribution function (ODF) [20-27]. Since clustering methods rely on the tractography results, they do not work properly if the tractography results are inaccurate. On the other hand, segmentation methods based on hyper-surface propagation do not use the tractography results and are thus more robust to noise.

In a region-based segmentation framework, a similarity measure between successive tensors is typically used. Some of the hyper-surface propagating methods in the literature concentrated on scalar quantities derived from the tensor data which do not reflect complete tensor information [20]. Other methods benefit from the entire information contained in the DTI data [21-30]. Wang and Vemuri [22] proposed a statistical level-set segmentation method. However, the tensors derived in this framework are not necessarily positive semi-definite, leading to inappropriate results especially when consecutive tensors are much different. Metrics like Kullback-Leibler divergence and J-divergence [23,24] have also been proposed. One of the most promising methods is introduced by Jonasson et al. [25]. They defined a new similarity measure called normalized tensor scalar product (NTSP). Comparing NTSP with other similarity measures, they demonstrated superiority of their proposed measure. To segment brain structures like thalamic nuclei, they modified their framework to favor the propagation of multiple hyper surfaces without overlapping [26]. Lenglet et al. [27] defined a dissimilarity measure and statistics between tensors based on the Riemannian distances. Although improving the segmentation results, this approach is computationally expensive. Defining a Log-Euclidean distance, another metric was defined by Arsigny et al. [28] which has lower computational burden. Weldeselassie and Hamarneh [29] used their proposed similarity measure in an energy minimization framework. Awate et al. [30] used the similarity measure in a Markov random field framework.

In terms of quantitative evaluation of diffusion parameters, previous studies have compared DTI-based indices in normal appearing white matter and corpus callosum in multiple sclerosis [4], stroke [5], schizophrenia [6], and Huntington's [7] and also studied the DTI methods to assess corpus callosum regions across the human lifespan [31]. For segmenting corpus callosum and its subdivisions in these studies, however, two-dimensional (2D) methods were applied and DTI-based indices compared in the mid-sagittal plane. However, without recruiting a three dimensional (3D) method to segment the whole corpus callosum and its subdivisions, the extracted quantities may be inaccurate.

Since the tensor model is not capable of describing heterogeneous diffusion behavior in the crossing fiber bundles, some studies used High Angular Resolution Diffusion Imaging (HARDI) data to segment specific bundles [32-38]. However, the HARDI data is not widely acquired in clinical centers and hence, the tensor-based methods are still of more practical use in clinical research.

In this manuscript, we present a 3D method to segment corpus callosum. Based on tensor and anisotropy values of neighboring voxels, a similarity measure is proposed and used as a speed function in the proposed level-set method. In this method, the principal diffusion direction (PDD) and prior information about the diffusivity pattern in corpus callosum are used to avoid inclusion of neighboring fiber bundles. Then, the Witelson subdivisions of corpus callosum are automatically identified [39]. The idea of using diffusivity pattern in corpus callosum has been used by Lee et al. [40]. However, they performed a 2D segmentation on the mid-sagittal plane. Moreover, since their method uses the left-right component of PDD to delineate corpus callosum boundaries, it is not applicable in more lateral sagittal planes, where corpus callosum connects to minor and major forceps and considerable anterior-posterior component of PDD exists.

The proposed segmentation method can be identically used for segmenting corpus callosum of control subjects as well as patients with glioblastoma. However, since we use the geometric information of the diffusivity pattern in corpus callosum and the glioblastoma tumor may change the original shape and diffusivity pattern of corpus callosum, we validate the reproducibility of the proposed method through realistic simulations of corpus callosum rotations under glioblastoma tumor pressure. We study the effects of rotation by different Euler angles (azimuth, elevation and skew) quantitatively and demonstrate that even in extreme cases with large rotations, brain tumors do not have major effects on the segmentation results generated by our proposed method. We apply the method to the DTI data of normal subjects and brain cancer patients with glioblastoma and show its superiority to some of the previously published methods in the literature.

## Methods

### Patient population

This study is approved by the institutional review board and is compliant with the Health Insurance Portability and Accountability Act (HIPAA). Between February 2006 and December 2008, 19 patients (8 males, 11 females; mean age 60.7 years) with treatment naïve glioblastoma underwent MRI with DTI using a 3 T scanner at our institution. Based on anatomical MRI findings, the patients were divided into two groups: Group 1 including patients with tumors not infiltrating corpus callosum (n = 12); and Group 2 including patients with tumors infiltrating corpus callosum (n = 7). For the control group, 11 patients (5 males, 6 females; mean age 48 years) who underwent brain MRI for nonspecific headache or single idiopathic seizure with normal MRI were included.

### Magnetic resonance imaging protocol

All the patients underwent both conventional MRI and DTI on a 3 T scanner (Excite HD, GE Medical Systems, Milwaukee, WI) using an 8-channel head coil. Diffusion weighted images (DWIs) were acquired in 25 diffusion gradient directions. The reconstructed DWIs have intra-slice resolution of 256 × 256 with voxel size of 0.98 × 0.98 × 2.5 mm. To have the same step size in each direction for the front propagation in the level-set method and to avoid extensive computation, we interpolated the data into 128 × 128 grid with cubical, 1.9 × 1.9 × 1.9 mm voxels.

### Level-set approach

We use a level-set method that takes into account several properties when formulating the segmentation problem [41,42]. The method smoothes the propagating hyper-surface automatically and leads to a regularized segmentation. Among the advantages of the method, its property of generalizing from 2D to 3D and higher dimensions and automatic splitting and merging of the surfaces are notable. In addition, it simplifies calculation of geometric quantities needed in the proposed method such as normal to surface and curvature.

For bundle segmentation in areas with high level of similarity in diffusion, the hyper-surface needs to grow in the direction normal to the surface. Moreover, the resulting fiber bundles must be smooth. Consequently, we use a level-set whose growing hyper-surface is the zero level-set of the following function which is a reduced form of the Hamilton-Jacobi partial differential equation:

(1) $$D_{t}\varphi \left(r,t\right)+F\left(r,t\right)||\nabla \varphi \left(r,t\right)||-\kappa \left(r,t\right)||\nabla \varphi \left(r,t\right)||=0$$

where r ∈ ℜ^n ^is the state space, φ: ℜ^n ^× ℜ → ℜ is the level-set function, D_t _φ is the partial derivative of φ with respect to the time variable *t*, ∇φ = D_r _φ is the gradient of φ with respect to the state space variables, and F(*r, t*) is the speed in the direction normal to the surface, extracted from the spherical harmonic coefficients of the neighboring voxels. The sign ∥·∥ stands for the magnitude operator. The curvature κ(*r, t*) is used to fulfill the smoothness constraint. To calculate the first order spatial partial derivative ∇φ(*r, t*), we use the 5th order of the upwind method [41].

### Corpus callosum segmentation

Corpus callosum is a commissural fiber bundle with a specific diffusion pattern which can be coded as prior knowledge in the segmentation framework. Using this information, we prevent the hyper-surface from propagating into adjacent white matter structures such as cingulum, tapetum, minor and major forceps, and tracts of the corona radiata. Although dissimilarity among tensors helps in this case, smooth and gradual transition in shape and direction of the DTI tensors from corpus callosum to minor and major forceps makes the segmentation difficult.

We define a similarity measure between every voxel on the propagating hyper-surface and its neighbors in the propagation direction, based on tensor and anisotropy values. This similarity measure is used as the speed term F(*r, t*) in Equation (1). The hyper-surface propagation in each step depends only on the speed function of the boundary voxels. This speed function term moves the hyper-surface to fill the whole fiber bundle, while the regularizing curvature term in Equation (1) is in charge of smoothing corpus callosum without changing its real structure.

Using the fact that the diffusivity in corpus callosum is perpendicular to the mid-sagittal plane of the brain, we consider a threshold (*PDD_x _Threshold*) for the x-component (left-right component) of the PDD (*PDD_x_(r*)) for propagating the front only inside the corpus callosum. The x-component of the PDD is large throughout the structure body. However, corpus callosum fibers project to the cortical area at its genu and splenium, where the x-component of PDD is not large anymore. Fortunately, these fibers are considered as different fiber tracts of minor and major forceps, and most of studies investigating the different diffusion indices within the corpus callosum, exclude the minor and major forceps from that.

In the proposed algorithm, we consider two more thresholds on collinearity of the PDD vectors (*Collinearity_Threshold*) and similarity of fractional anisotropy (FA) values (*FA_Threshold*) in the neighboring voxels.

The proposed segmentation steps are as follows:

1. Select the initial seeds in corpus callosum in mid-sagittal plane manually.

2. Initiate the hyper-surface as the congregation of small spheres around the seed points.

3. Do until convergence

• For each point *r *on the hyper-surface at step *t*:

a) Calculate the normal direction to the surface.

b) Calculate the 26-neighborhood and keep the neighbors *n_r _*for *r*, which are collinear with the normal, with respect to the *r*.

c) If *PDD*(*r*). *PDD*(*n_r_*) >*Collinearity_Threshold & FA *(*n_r_*) >*FA_Threshold & PDD*_x _(*r*) >*PDD_x__Threshold*

(2) $$\text{Then}\;F\left(r,t\right)=\underset{n_{r}}{\sum }FA\left(r\right).FA\left(n_{r}\right).\frac{tr\left[D{\left(r\right)}^{*}D\left(n_{r}\right)\right]}{tr{\left[D\left(r\right)\right]}^{*}tr\left[D\left(n_{r}\right)\right]}$$

• Threshold *F*(*r, t*) with *F _ Threshold *to diminish the effect of negligible speeds.

• Use the resultant speed in the level-set framework.

4. Extract the zero level-set as the segmented corpus callosum.

In Equation (2), *tr(.) *is the matrix trace and *D(r) *is the tensor at point *r*.

After segmenting corpus callosum, Witelson subdivisions of corpus callosum are automatically extracted [39]. First, the critical point between genu and rostrum of corpus callosum is calculated, where the curvature of the structure boundary in mid-sagittal plane changes. Then, the segmented corpus callosum is automatically subdivided into Witelson subdivisions in the mid-sagittal plane: rostrum, genu, rostral body, anterior mid-body, posterior mid-body, isthmus and splenium [39]. Moreover, the user can visualize and confirm the calculated mid-sagittal plane and the critical joining point between genu and rostrum. The critical point can be selected manually if close supervision is preferred or needed.

## Results

### Segmentation using proposed method

We implemented the proposed algorithm in MATLB R2008a using a PC with Intel^® ^Core™ 2Duo CPU (E8400@ 3.00 GHz, 3.00 GHz) and 4 GByte RAM and 64 bit VISTA operating system.

To evaluate quality of the segmentation results, the dice correctness measure [43] is calculated using the following relation:

(3) $$Correctness=\frac{N\left(S_{a}\cap S_{y}\right)}{\left[N\left(S_{a}\right)+N\left(S_{y}\right)\right]/2}$$

where S_a _and *S_r _*are the automatic and manual (reference) segmentation results, respectively, and *N *is the number of voxels in each bundle. Here, N(S_a _∩ S_y_)is the number of the True-Positives. The Dice correctness measure is appropriately bounded, normalized, well-understood, and applied widely in evaluating segmentation methods. Two of the co-authors (a clinical expert and a technical expert) sat down together and carried out the manual segmentation which is considered as the reference segmentation results.

**Figure 1:**
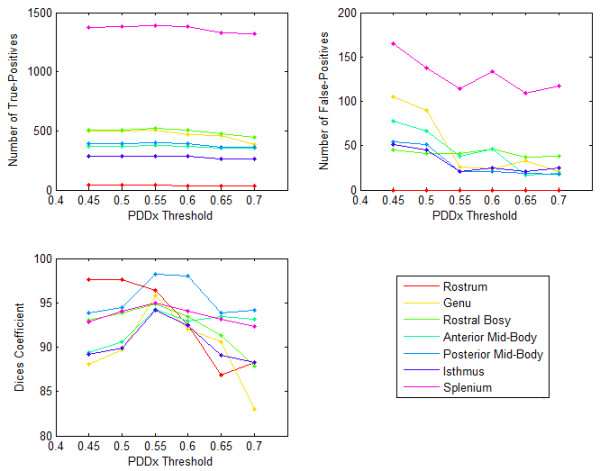
**Sensitivity of corpus callosum Witelson segments to PDD_x_Threshold for a normal subject**. The graphs show number of True-Positives, number of False-Positives, and Dice correctness measure over a range of PDD_x_Threshold for Witelson segments of the Corpus Callosum.

**Figure 2:**
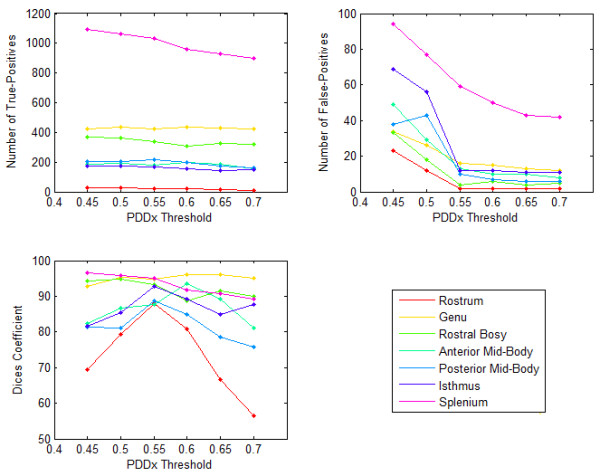
**Sensitivity of corpus callosum Witelson segments to PDD_x_Threshold for a tumor patient**. The graphs show number of True-Positives, number of False-Positives, and Dice correctness measure over a range of PDD_x_Threshold for Witelson segments of the Corpus Callosum.

We tested sensitivity of corpus callosum Witelson segments to *PDD_x__Threshold *for a normal subject. Figures 1 and 2 show the number of True-Positives, the number of False-Positives, and the Dice correctness measure over a range of *PDD_x__Threshold *for the Witelson segments of the Corpus Callosum of a normal subject and a tumor patient, respectively. As shown in these figures, for *PDD_x__Threshold *values within a specific range (here 0.5 to 0.6), the Dice correctness measure is maximized. For a higher *PDD_x__Threshold*, both the number of True-Positives and False-Positives start to decrease, however, the number of False-Positives tend to saturate by increasing the *PDD_x__Threshold*. This occurs conversely for *PDD_x__Threshold *values lower than a specific range, i.e., both the number of True-Positives and False-Positives start to increase, however, the number of True-Positives tends to saturate by decreasing *PDD_x__Threshold*. As seen, there is a small difference between the optimal selection of *PDD_x__Threshold *for the normal subject and the tumor case, suggesting that we can apply the same *PDD_x__Threshold *for all datasets.

**Figure 3:**
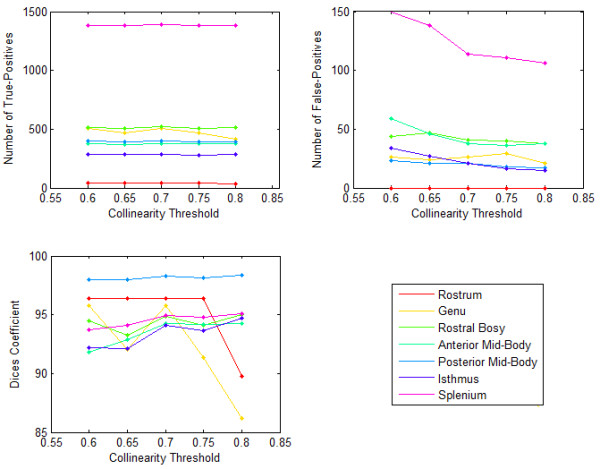
**Sensitivity of corpus callosum Witelson segments to Collinearity _Threshold for a normal subject**. The graphs show number of True-Positives, number of False-Positives, and Dice correctness measure over a range of Collinearity _Threshold for the Witelson segments of the Corpus Callosum of a normal subject.

Figure 3 shows the number of True-Positives, the number of False-Positives, and the Dice correctness measure over a range of *Collinearity _Threshold *for the Witelson segments of the Corpus Callosum of a normal subject. It is shown that when *Collinearity _Threshold *values are in a specific range (here 0.65 to 0.75), the Dice correctness measure is maximized. However, the sensitivity of the segmentation method to *Collinearity _Threshold *is less than its sensitivity to *PDDx_Threshold*. We found the following set of parameters optimal for segmenting corpus callosum: PDD_x__Threshold = 0.55, Collinearity _Threshold = 0.7, *FA_Threshold *= 0.1, and *F_Threshold *= 0.05.

**Figure 4:**
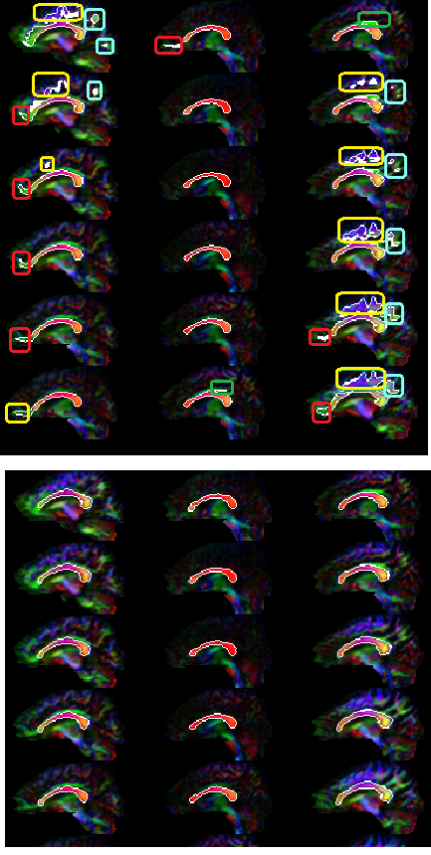
**Comparing of corpus callosum segmentations by the proposed method and the Jonasson's method **[25]. In (**a**) for the Jonasson's method, the speed threshold was chosen low enough to capture the corpus callosum structure. However, the segmentation front propagates inside irrelevant fiber structures such as superior longitudinal fasciculus (cyan rounded rectangles), cingulum (green rounded rectangles), minor forceps (red rounded rectangles), and tracts of corona-radiata (yellow rounded rectangles). In (**b**), our method segments the corpus callosum structure with high sensitivity (segmenting the whole structure) and high specificity (without major leakage into the neighboring structures).

The tensor-based method proposed by Jonasson et al. [32] with NTPP similarity measure is quite sensitive to the speed threshold. If the speed threshold is selected high enough to prevent the front from propagating into the neighboring structures, corpus callosum is not segmented entirely (low sensitivity). However, if the speed threshold is chosen low enough to segment the entire corpus callosum, the front propagates into irrelevant fiber structures such as superior longitudinal fasciculus, cingulum, minor forceps, and tracts of corona-radiata (low specificity). Figure 4a shows the results of our implementation of their method. In this figure, we tuned the speed threshold so that the whole structure is segmented. Figure 4b demonstrates high sensitivity (segmenting almost the whole structure) and high specificity (without major penetration into the neighbouring structures) of our proposed method compared to the Jonasson's method.

We have also compared the results of the proposed method with those of the Jonasson's method quantitatively. Table 1 shows the average Dice measures for different Witelson subdivisions of corpus callosum for the control subjects. Note that the best performance is obtained in the genu area with least penetration inside adjacent fiber bundles and the worst performance is obtained in the posterior mid-body subdivision with penetration mainly inside cingulum and tracts of corona-radiata. For the tumor patients, the performance of the Jonasson's method was poor and thus we do not present them.

**Table 1:** Comparison of the proposed method with the Jonasson's method

Witelson Subdivisions	Proposed Method	Jonasson's Method
**Rostrum**	94.41	75.15

**Genu**	98.25	91.24

**Rostral Body**	97.27	83.10

**Anterior Mid-Body**	95.44	79.33

**Posterior Mid-Body**	96.07	71.54

**Isthmus**	94.24	81.46

**Splenium**	97.29	80.25

The average Dice measures for different Witelson subdivisions of the corpus callosum for the control subjects. Note that the best performance is obtained in the genu area with least penetration inside adjacent fiber bundles, and the worst performance is obtained in the posterior mid-body subdivision with penetration mainly inside cingulum and tracts of corona-radiata.

Table 2 shows the average Dice measure for different Witelson subdivisions of corpus callosum in the control subjects, Group 1 patients (tumor not infiltrating corpus callosum), and Group 2 patients (tumor infiltrating corpus callosum). Dice measures ranging from 90% to 98% indicate that the automatic segmentation results generated for the control subjects and the Group 1 patients are in excellent agreement with the manual segmentation results. For Group 2 patients where the tumors infiltrated corpus callosum, the Dice measures ranging from 81% to 92% indicate good agreements of the automatic and manual segmentation results.

**Table 2:** Average Dice measures for different Witelson subdivisions of the corpus callosum

Witelson Subdivisions	Control Subjects	Group 1 Patients	Group 2 Patients
**Rostrum**	94.41	94.04	90.72

**Genu**	98.25	94.99	92.61

**Rostral Body**	97.27	94.05	84.73

**Anterior Mid-Body**	95.44	91.45	81.58

**Posterior Mid-Body**	96.07	94.1	83.29

**Isthmus**	94.24	94.57	85.09

**Splenium**	97.29	90.16	89.56

The subjects are classified to control subjects, Group 1 patients with tumors not infiltrating the corpus callosum, and Group 2 patients with tumors infiltrating the corpus callosum. Note that the high quality of the results for the control subjects as well as the tumor patients.

**Figure 5:**
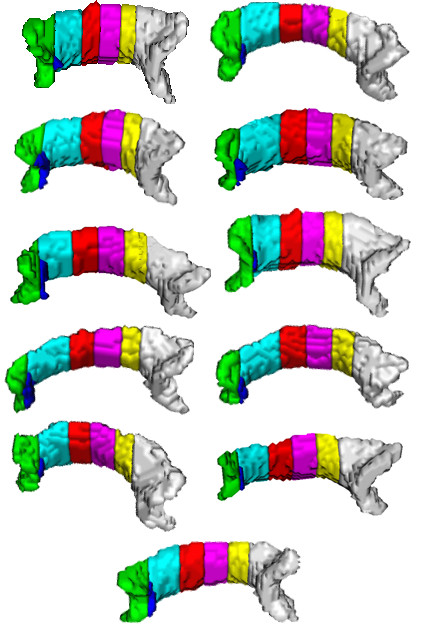
**Segmented corpus callosum by the proposed method for 11 control subjects**. The colors show the Witelson subdivisions results for the corpus callosum. Rostrum: blue, Genu: green, Rostral Body: cyan, Anterior Mid-body: red, Posterior Mid-body: turquoise, Isthmus: yellow, Splenium: gray.

**Figure 6:**
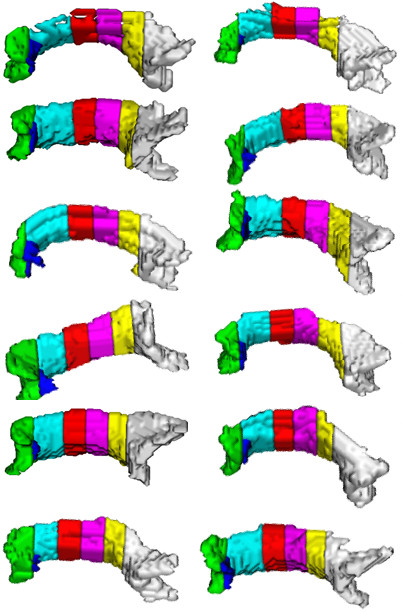
**Segmented corpus callosum by the proposed method for 12 glioblastoma patients with non-infiltrating tumors**. The colors show the Witelson subdivisions results for the corpus callosum. Rostrum: blue, Genu: green, Rostral Body: cyan, Anterior Mid-body: red, Posterior Mid-body: turquoise, Isthmus: yellow, Splenium: gray.

**Figure 7:**
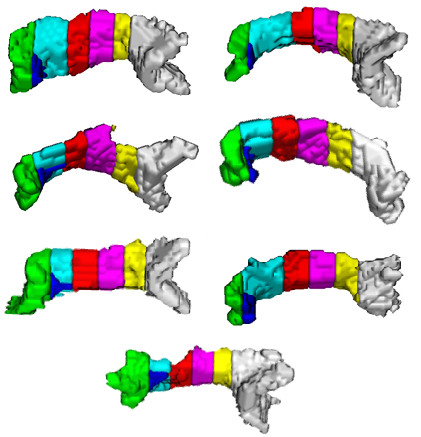
**Segmented corpus callosum by the proposed method for 7 glioblastoma patients with infiltrating tumors**. The colors show the Witelson subdivisions results for the corpus callosum. Rostrum: blue, Genu: green, Rostral Body: cyan, Anterior Mid-body: red, Posterior Mid-body: turquoise, Isthmus: yellow, Splenium: gray.

Performance of the proposed method can be ascertained by visualizing the segmentation results (Figures 5, 6, 7). Figure 5 shows the segmentation results of the proposed method for 11 control subjects. Figure 6 shows the results for 12 Group 1 patients where the glioblastoma tumor does not infiltrate corpus callosum. These 12 patients were selected based on the severity of the effect of tumor on the rotation of corpus callosum. Note that although these cases include major geometric deviations from the normal state, the proposed method has successfully segmented corpus callosum and its subdivisions. Figure 7 shows the segmentation results for 7 Group 2 patients where the glioblastoma tumor has infiltrated corpus callosum. This figure illustrates that the proposed method has successfully segmented corpus callosum and its subdivisions in these extreme cases. Note that in all cases, the segmented corpus callosum does not penetrate into the adjacent fiber bundles such as cingulum, tapetum, minor and major forceps, superior longitudinal fasciculus, or tracts of corona radiata.

### Rotational effect of tumor on proposed method

We have used the geometric information of the diffusivity pattern in corpus callosum to prevent the front from penetrating into the neighboring structures. However, a tumor may change the shape and diffusivity pattern of corpus callosum from its original shape and diffusivity pattern.

To study the rotational effect of a tumor on the segmentation process, we rotate the segmented corpus callosum and its neighbors up to 5 voxels in different Euler angles (*azimuth*-*elevation*-*skew*), the tensor (T), and the principal diffusion direction (PDD) of each voxel for all control subjects.

(4) $$R\left(\varphi ,\theta ,\psi \right)=R_{x}\left(\varphi \right)R_{y}\left(\theta \right)R_{z}\left(\psi \right)$$

where R(*φ,θ,ψ*) is the rotation matrix with respect to the spherical Euler angles of *φ,θ *and *ψ *respectively. R _x _(φ), *R_y _*(*θ*), and *R_z _*(*ψ*) are rotational matrices for rotations around *x, y*, and *z *axes, respectively. Then,

(5) $$\left(x^{\prime },y^{\prime },z^{\prime }\right)=R{\left(\varphi ,\theta ,\psi \right)}^{*}\left(x,y,z\right)$$

where (x, y, z) is the coordinates for a voxel within a 5-voxel neighborhood of corpus callosum, while (x', y'z') is the rotated voxel coordinates.

For each of the rotated coordinates, we calculate the tensor (T) and the principal diffusion direction (PDD) by:

(6) $$T\left(x^{\prime },y^{\prime },z^{\prime }\right)={\text{R}}^{\text{t}}\text{(}\phi \text{,}\theta \text{,}\psi \text{)*T(x,y,z)*R(}\phi \text{,}\theta \text{,}\psi \text{)}$$

(7) $$PDD\left(x^{\prime },y^{\prime },z^{\prime }\right)=\text{R(}\phi \text{,}\theta \text{,}\psi \text{)*PDD(x,y,z)}$$

The means and standard deviations of the Dice measures for the subdivisions of the corpus callosum rotated in different Euler angles (5, 10, 15, 20, 25, and 30 degrees) in 11 control subjects are shown in Table 3. The top, middle, and bottom rows represent the segmentation results of the rotated corpus callosum and its neighbors under different azimuth angles (around *x *axis), elevation angles (around *y *axis), and skew angles (around *z *axis), respectively. As can be seen from the Tables 2 and 3, the rotation of corpus callosum does not have a major effect on the segmentation results. It means that although the segmentation algorithm relies on the geometric information of corpus callosum and its diffusion pattern, it segments a corpus callosum deformed under the potential tumor pressure.

**Table 3:** Means and standard deviations of the Dice measures in 11 normal subjects where corpus callosum subdivisions are rotated under different Euler angles

Witelson Subdivisions of Corpus Callosum		Rostrum	Genu	Rostral Body	Anterior Mid-Body	Posterior Mid-Body	Isthmus	Splenium
**Azimuth Rotation Angle**	**5**	94.51 ± 1.15	96.46 ± 1.43	99.07 ± 0.84	98.48 ± 0.77	99.28 ± 0.56	96.84 ± 0.91	96.23 ± 0.93
	
	**10**	91.44 ± 0.99	95.48 ± 0.98	95.02 ± 1.13	95.45 ± 0.99	97.36 ± 1.03	96.27 ± 0.93	94.11 ± 1.01
	
	**15**	91.00 ± 1.47	90.77 ± 1.15	94.17 ± 0.95	94.47 ± 0.96	94.97 ± 0.70	92.84 ± 1.01	90.55 ± 0.97
	
	**20**	88.29 ± 1.24	89.33 ± 1.07	89.65 ± 0.75	90.61 ± 1.23	88.86 ± 0.86	89.18 ± 1.23	88.40 ± 1.06
	
	**25**	84.24 ± 1.03	88.47 ± 1.12	89.16 ± 0.86	87.32 ± 1.03	88.83 ± 1.06	89.01 ± 1.49	84.69 ± 1.20
	
	**30**	80.16 ± 1.70	82.96 ± 1.23	85.09 ± 1.21	85.45 ± 1.13	85.36 ± 0.98	83.07 ± 1.11	81.23 ± 0.97

**Elevation Rotation Angle**	**5**	93.64 ± 0.92	96.29 ± 1.28	97.75 ± 0.97	96.91 ± 1.02	98.65 ± 1.16	96.29 ± 0.72	95.83 ± 1.09
	
	**10**	91.27 ± 0.88	96.24 ± 1.08	97.74 ± 1.00	95.46 ± 1.06	94.14 ± 1.09	91.56 ± 1.17	91.96 ± 1.41
	
	**15**	87.75 ± 0.86	95.28 ± 1.15	93.86 ± 0.89	93.81 ± 0.97	94.95 ± 0.83	89.30 ± 0.91	91.08 ± 1.14
	
	**20**	85.37 ± 1.01	92.58 ± 0.77	90.59 ± 0.89	89.98 ± 1.29	92.51 ± 1.05	87.36 ± 1.32	86.29 ± 0.88
	
	**25**	83.47 ± 0.90	86.67 ± 0.77	86.25 ± 1.32	89.27 ± 1.17	87.66 ± 0.99	84.88 ± 1.27	84.48 ± 1.10
	
	**30**	79.39 ± 1.29	81.60 ± 1.10	85.00 ± 1.10	84.35 ± 1.01	85.22 ± 1.03	83.41 ± 0.88	81.58 ± 0.89

**Skew Rotation Angle**	**5**	97.96 ± 1.09	98.75 ± 0.63	99.09 ± 0.68	98.00 ± 1.07	98.73 ± 1.00	96.62 ± 0.73	98.85 ± 0.59
	
	**10**	95.29 ± 1.12	96.38 ± 0.80	94.72 ± 1.04	94.46 ± 1.45	94.80 ± 1.28	94.98 ± 1.00	94.21 ± 0.88
	
	**15**	91.88 ± 1.04	95.00 ± 1.34	92.56 ± 1.16	94.22 ± 0.97	92.73 ± 1.31	94.72 ± 1.44	93.66 ± 1.01
	
	**20**	89.79 ± 0.83	87.92 ± 1.18	85.83 ± 1.29	88.35 ± 1.16	86.99 ± 1.27	87.85 ± 0.98	89.28 ± 1.16
	
	**25**	87.55 ± 1.22	86.11 ± 1.21	85.73 ± 1.08	86.34 ± 1.28	86.22 ± 1.08	85.55 ± 1.27	87.35 ± 0.87
	
	**30**	84.61 ± 1.17	83.39 ± 0.89	84.58 ± 1.13	84.09 ± 1.03	85.26 ± 1.12	86.43 ± 1.16	85.00 ± 1.00

It can be inferred that the rotation of corpus callosum does not have a major effect on the segmentation results.

**Figure 8:**
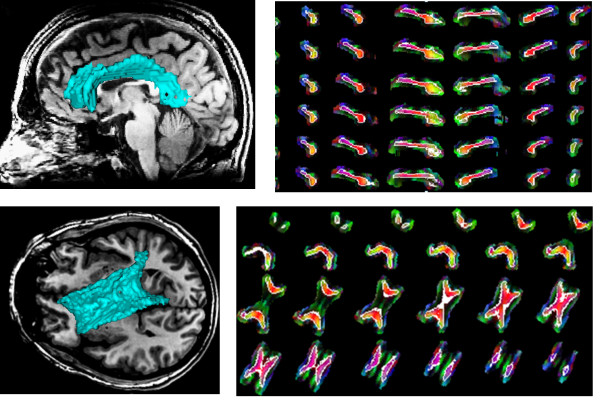
**Segmentation results by the proposed method for a normal corpus callosum and its neighbors rotated 30° under the azimuth angle**. (**a**), (**b**): Segmented corpus callosum in green overlaid on the T1 sagittal and axial images, respectively. (**c**), (**d**): Boundaries of the corpus callosum delineated in white in the sagittal and axial slices, respectively, overlaid on the color coding of the principal diffusion direction in each pixel.

**Figure 9:**
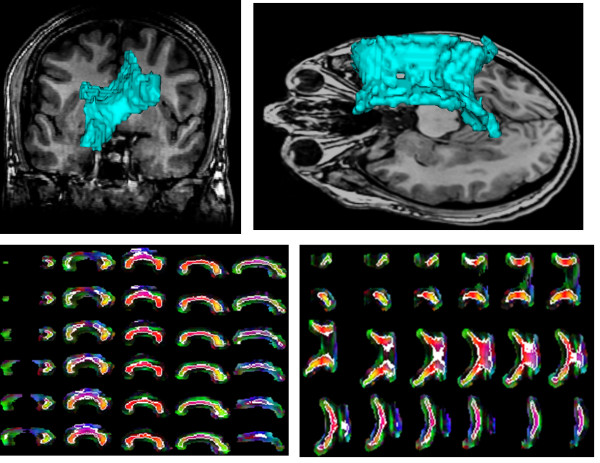
**Segmentation results by the proposed method for a normal corpus callosum and its neighbors rotated 30° under the elevation angle**. (**a**), (**b**): Segmented corpus callosum in green overlaid on the T1 sagittal and axial images, respectively. (**c**), (**d**): Boundaries of the corpus callosum delineated in white in the sagittal and axial slices, respectively, overlaid on the color coding of the principal diffusion direction in each pixel.

**Figure 10:**
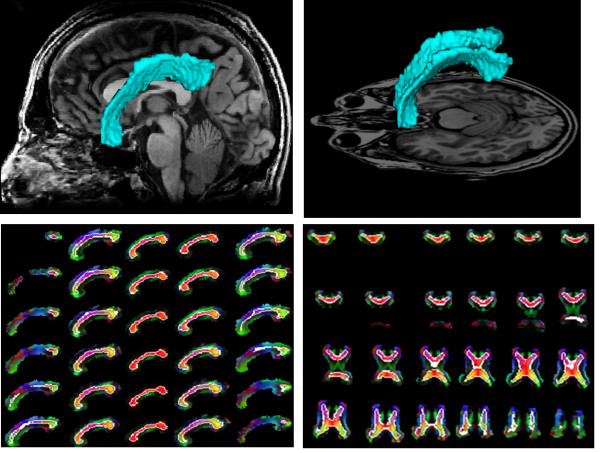
**Segmentation results by the proposed method for a normal corpus callosum and its neighbors rotated 30° under the skew angle**. (**a**), (**b**): Segmented corpus callosum in green overlaid on the T1 sagittal and axial images, respectively. (**c**), (**d**): Boundaries of the corpus callosum delineated in white in the sagittal and axial slices, respectively, overlaid on the color coding of the principal diffusion direction in each pixel.

We used the Wilcoxon two sample tests to compare sensitivity of the outer subdivisions (rostrum, genu, and splenium) and the inner subdivisions (rostral body, anterior and posterior mid-body, and isthmus) of corpus callosum with respect to the rotation. For two arrays *A *and *B*, the Wilcoxon test performs a paired two-sided signed rank test of the null hypothesis that data in the vector *A-B *come from a symmetric distribution with zero median. P-values less than 0.01 are considered statistically significant. From the p-values in Table 4, it can be inferred that the outer subdivisions have significantly lower average Dice measures than the inner subdivision for all azimuth and elevation rotation angles (*p*-values less than 0.0004). For skew angles, however, the outer subdivisions do not show significantly lower average Dice measures than the inner subdivision (5 out of 6 p-values are more than 0.01). Considering the overall diffusivity inside corpus callosum perpendicular to its main axis, the overall diffusivity pattern changes more dramatically around the *x *and *y *axes (azimuth and elevation) than around the *z *axis (skew). Therefore, the segmentation results are more sensitive to rotations in the azimuth and elevation directions. Figures 8, 9 and 10 show the segmentation results of the proposed method for the corpus callosum and its neighbors rotated 30° in the azimuth, elevation, and skew directions, respectively.

**Table 4:** The *p*-value of Wilcoxon two-sample tests between outer (rostrum, genu, and splenium) and inner (rostral body, anterior and posterior mid-body, and isthmus) subdivisions of corpus callosum

	Azimuth Rotation Angle					
	5	10	15	20	25	30

***P*-value**	8.2E-05	8.1E-05	8.1E-05	7.1E-03	8.2E-05	8.2E-05

	**Elevation Rotation Angle**					

	5	10	15	20	25	30

***P*-value**	8.2E-05	3.9E-04	1.4E-04	8.2E-05	8.0E-05	8.2E-05

	**Skew Rotation Angle**					

	5	10	15	20	25	30

***P*-value**	7.6E-02	8.2E-02	9.2E-01	2.4E-04	5.8E-03	3.6E-02

The outer subdivisions have significantly lower average Dice measures than the inner subdivision for all azimuth and elevation rotation angles (p-values less than 0.0004). For skew angles, however, the outer subdivisions do not show significantly lower average Dice measures than the inner subdivision (5 out of 6 p-values are more than 0.01). See Table 3 for the means and standard deviations of the 11 subjects.

## Discussion

### Novel aspects of proposed method

#### 1. New similarity measure based on local diffusion characteristics

The proposed method is more accurate than the tensor-based method that uses the NTSP similarity measure [25]. Comparing with the manual segmentation by experts, we demonstrated accuracy of our proposed method even when the tumor infiltrates corpus callosum.

#### 2. 3D segmentation of the entire corpus callosum

The proposed method works in 3D. Extraction of diffusion indices such as mean diffusivity and fractional anisotropy over the entire corpus callosum generates a reliable quantification of the structure that cannot be achieved by an analysis of the mid-sagittal plane only [4-7].

#### 3. Preventing from penetrating inside neighboring fiber bundles

An optimal set of parameters is chosen to segment the entire corpus callosum (with high sensitivity) without penetrating into adjacent fiber structures (with high specificity). We have used *Collinearity_Threshold *to prevent the front from penetrating into cingulum. The threshold for the x-component of the principal diffusion direction (*PDD_x__Threshold*) is used to prevent the front from propagating into major and minor forceps. *FA_Threshold *is also important to prevent from penetrating inside tapetum (as in general, it has diffusivity patterns similar to corpus callosum in crossing areas with lower FA values). To segment the entire corpus callosum in patients with low FA values, one should choose *FA_Threshold *and *F_Threshold *quite small. However, one should be cautious that in this case, the front may propagate outside of white matter in some areas.

#### 4. Automatically extracting Witelson subdivisions of corpus callosum

The proposed method defines Witelson subdivisions of corpus callosum automatically.

#### 5. Applicability to glioblastoma tumor patients as well as normal subjects using the same set of parameters

The proposed method has successfully segmented corpus callosum and its subdivisions in diffusions MRI data of normal subjects and brain tumor patients.

#### 6. Evaluating potential effects of tumor on segmentation of corpus callosum

Depending on size, shape, type and proximity to the corpus callosum, the glioblastoma tumor may cause rotation, shrinkage or more severe disruptions like tearing of the corpus callosum fibers. Amongst the mentioned effects, we evaluated the linear rotational effect of tumor on corpus callosum. With this simplification and without loss of generality, we showed that the change in diffusivity pattern due to a tumor does not change the segmentation accuracy dramatically. However, if the effect of tumor infiltration inside the corpus callosum is sever and changes the structure dramatically, it may be impossible to segment the structure entirely and accurately.

### Selection of initial seeds

The proposed segmentation method is seed-based and needs the initial seed points for hyper-surface to propagate. This requires the operators to define the seeds manually using anatomical landmarks. To automate the process, the initial seeds may be obtained from fiber atlases of control subjects [34,44]. However, when segmenting fiber bundles of pathological cases, abnormalities like tumors may change the fiber bundles and thus conventional atlas registration methods may not be applicable. To solve this problem, Zacharaki et al. [45] proposed a method for transferring structural and functional information from neuro-anatomical brain atlases into individual patient's data. Application of such methods in our case of segmenting corpus callosum in patients with tumors would be still in question.

### Segmentation of fiber structures in HARDI

Since the tensor model is not capable of describing heterogeneous diffusion behavior in crossing fiber bundles, some studies segmented the desired fiber bundles using HARDI data. Generalizing the level-set method presented in [22] to the HARDI data, Descoteaux and Deriche [35] applied a region-based statistical surface evolution to the image of the ODFs to find coherent white matter fiber bundles. This is equivalent to the maximization of a posteriori probability which obtains the desired segmentation for the observed ODFs and presumes Gaussian distributions in different partitions of the Q-ball images. Although their method appropriately propagates through the regions of fiber crossings, it propagates the hyper-surface inside all of the crossing fiber bundles and segments them as a whole, not individually. They applied their method to extract corpus callosum and tracts of corona-radiata. However, their method is sensitive to initial seeds and suffers from limited anatomical knowledge of the operator who defines the seeds. In another study, a k-means algorithm has been employed to find the clusters using Euclidean distance as dissimilarity measure [36].

In a different study, a Position-Orientation Space (POS) is introduced by combining the geometric space with the spherical ODF space from the HARDI data, where two crossing fiber populations with different orientations in the spatial domain are resolved by applying a front propagation method in the level-set framework in a five-dimensional space [32]. Hagmann et al. [36] performed fiber bundle segmentation in POS based on the Markov Random Fields (MRF). In a similar study, McGraw et al. [37] proposed a mixture of von Mises-Fisher distributions to model the ODF again in MRF. Assuming that the spatial relationships are modeled by the MRF, this method estimates a hidden random field of fiber bundles from the observed ODF profiles using a Maximum a Posteriori (MAP) formulation. However, dimensional reinforcement of the problem causes disadvantages such as increasing the computational cost.

Using Spherical Harmonic Coefficients (SHC) as features of functions on the sphere [38], a method has been proposed for fiber bundle segmentation [33]. However, without masking the speed term with a measure of anisotropy (such as FA) that has low values in the crossing areas, the growing hyper-surface may penetrate into irrelevant fiber bundles that have common areas with the bundle of interest. In another work [34], the authors proposed an atlas-based method introducing a novel similarity based on PDD's and spherical harmonic coefficients. Integrating PDD's into the framework, along with a proper PDD-selection algorithm, leads to the segmentation of most of important fiber bundles in the brain without penetration into irrelevant fiber bundles that have common areas with the bundle of interest. Using an atlas [44] to find the initial seeds for the fiber bundles, the proposed method overcomes limitations of the semi-automatic methods that suffer from limited anatomical knowledge and subjectivity of the operator who defines the seed voxels. Note that since the number of diffusion measurements in the tensor data is not adequate for fitting SHC and estimating more than one PDD, we used the same methods originally applied on the HARDI data.

Although the success of recent studies in segmenting fiber bundles in the HARDI data is promising, such protocol in not being widely used in clinical centers. On the other hand, diffusion tensor imaging is clinically feasible and thus the tensor-based methods are of more interest.

## Conclusion

In this paper, we have proposed a 3D method based on a new DTI similarity measure to segment corpus callosum and determine its subdivisions. The method propagates a hyper-surface within corpus callosum without penetration into the neighboring fiber bundles. Segmentation of corpus callosum, the largest commissural fiber bundle in the brain, makes it possible to quantify various diffusion characteristics in its subdivisions, opening new perspectives for monitoring disease evolution or prognosis.

### Ethics statement

MRI and other data of the tumor patients originally acquired for patient care are retrospectively and anonymously used in this research to train, test, and evaluate the proposed methods. This usage was reviewed and approved by the Institutional Review Board (IRB) committee of Henry Ford Hospital, Detroit, Michigan, USA. Details of the data and results are described in the manuscript.

## Competing interests

The authors declare that they have no competing interests.

## Authors' contributions

MN implemented the algorithms presented in the manuscript, tested the algorithms, and prepared the first draft of the manuscript. RJ participated in the design and coordination of the study, helped with the testing and evaluation of the proposed method, and revised the manuscript. SS contributed to the manual segmentation, revision of the manuscript, and final evaluation of the results. AB performed the statistics on the segmentation results. HS contributed to the revision of the manuscript and analysis of the results. MR, QJ, TM contributed to the image acquisition, selection of the cases, and classifying them into different groups. All authors read and approved the final manuscript.

## Pre-publication history

The pre-publication history for this paper can be accessed here:

http://www.biomedcentral.com/1471-2342/12/10/prepub
